# Activation of an early feedback survival loop involving phospho-ErbB3 is a general response of melanoma cells to RAF/MEK inhibition and is abrogated by anti-ErbB3 antibodies

**DOI:** 10.1186/1479-5876-11-180

**Published:** 2013-07-27

**Authors:** Luigi Fattore, Emanuele Marra, Maria Elena Pisanu, Alessia Noto, Claudia de Vitis, Francesca Belleudi, Luigi Aurisicchio, Rita Mancini, Maria Rosaria Torrisi, Paolo Antonio Ascierto, Gennaro Ciliberto

**Affiliations:** 1 Dipartimento di Medicina Clinica e Molecolare, Sapienza Università di Roma, Rome, Italy; 2 Dipartimento di Chirurgia “P. Valdoni”, Sapienza Università di Roma, Rome, Italy; 3 Takis srl, Rome, Italy; 4 Dipartimento di Medicina Sperimentale e Clinica, Università degli Studi di Catanzaro “Magna Graecia”, Catanzaro, Italy; 5 Istituto Pasteur Fondazione Cenci Bolognetti, Dipartimento di Medicina Clinica e Molecolare, Sapienza Università di Roma, Rome, Italy; 6 Azienda Ospedaliera S. Andrea, Rome, Italy; 7 Istituto Nazionale per lo Studio e la Cura dei Tumori “Fondazione G. Pascale”, Via Mariano Semmola, 80131 Naples Italy

**Keywords:** Melanoma, BRAF/MEK inhibitors, ErbB3 hyperphosphorylation, Anti-ErbB3 antibodies

## Abstract

**Background:**

Treatment of advanced melanoma has been improved with the advent of the BRAF inhibitors. However, a limitation to such treatment is the occurrence of resistance. Several mechanisms have been identified to be responsible for the development of resistance, either MEK-dependent or MEK-independent. In order to overcome resistance due to reactivation of MEK signaling, MEK inhibitors are being clinically developed with promising results. However, also in this case resistance inevitably occurs. It has been recently reported that ErbB3, a member of the EGFR receptor family, may be involved in the establishment of drug resistance.

**Methods:**

Three melanoma cell lines were tested: LOX IMVI (BRAF V600E), MST-L (BRAF V600R) and WM266 (BRAF V600D). Phosphorylation of Receptor Tyrosine Kinases (RTKs) was assessed by an RTK array. Western blot analysis was performed on total protein extracts using anti-ErbB3, anti-AKT and anti-ERK 1/2 antibodies. The expression of neuregulin after vemurafenib treatment was assessed by Real Time PCR and Western blotting. The growth inhibitory effects of vemurafenib, GSK1120212b and/or anti-ErbB3 mAbs were evaluated by *in vitro* colony formation assays.

**Results:**

In the present study we demonstrate that ErbB3 is the main RTK undergoing rapidly hyperphosphorylation upon either treatment with a BRAF inhibitor or with a MEK inhibitor in a panel of melanoma cell lines harboring a variety of V600BRAF mutations and that this results in a strong activation of phospho-AKT. Importantly, ErbB3 activation is fully abrogated by the simultaneous use of anti-ErbB3 monoclonal antibodies, which are also shown to potently synergize with BRAF inhibitors in the inactivation of both AKT and ERK pathways and in the inhibition of melanoma cell growth. We show that upregulation of phospho-ErbB3 is due to an autocrine loop involving increased transcription and production of neuregulin by melanoma cells.

**Conclusions:**

On the basis of these results, we propose that initial co-treatment with BRAF and/or MEK inhibitors and anti-ErbB3 antibodies should be pursued as a strategy to reduce the ErbB3-dependent feedback survival mechanism and enhance duration of clinical response.

## Background

Malignant melanoma is the deadliest form of skin cancer. Over the last 60 years there has been an approximate 700% increase in the incidence of melanoma world-wide and mortality has also increased by 165% [1]. Disease-specific survival curves in all stages of melanoma have a negative slope and overall prognosis is poor with less that 5% of stage 4 patients surviving 5 years from the manifestation of metastatic disease [1]. Before 2011 only three drugs were FDA-approved for metastatic melanoma, fotemustine, dacarbazine and high-dose IL2, all of them giving rise to modest response rates (6 to 15%) with median progression free survival (PFS) of 1.7 months, only 25.5% of patients still alive at 1 year and rare long-term regressions [2]. In recent years however, the scenario has completely changed thanks to the development of innovative systemic therapies. In first instance immunotherapy with ipilumumab has demonstrated improved survival in patients with advanced melanoma in Phase III randomized trials [3]. At the same time novel agents directed to target cell autonomous disregulated pathways have shown remarkable clinical effects. The first one is vemurafenib, a selective inhibitor of BRAF-activating mutations which are found in more than 50% of melanomas and which cause constitutive activation of the MAPK/ERK pathway driving uncontrolled melanoma growth. In a first Phase I trial vemurafenib (previously called PLX4032) achieved an objective response rate in excess of 50-60% in advanced disease [4]. Subsequently a phase III study comparing vemurafenib to dacarbazine showed a significant increase in survival for patients receiving vemurafenib [5]. Other more potent BRAF inhibitors are in advanced clinical development, having achieved promising results in early trials [6]. It is important to point out that BRAF inhibitors are active only in tumors where V600 BRAF mutations result in constitutively active monomers, whereas the same inhibitors give rise to paradoxical tumor promoting effects in RAS mutated melanomas because of their ability to induce allosteric activation through homo- or hetero-dimerization of wild type RAF isoforms [7,8]. Hence, the current strategy to tackle NRAS mutated melanomas involves the use of inhibitors of more downstream kinases in the RAS-RAF-MAPK pathway, in particular MEK [9]. In line with this, very recently MEK inhibitors have shown clinical activity as single agents in patients bearing mutated NRAS [10].

Although BRAF inhibitors (BRAFi) induce unprecedented objective responses virtually all responders suffer from disease progression due to the development of de novo drug resistance [11,12]. Thus, in order to significantly improve melanoma survivability it is necessary to develop new approaches to overcome or, better, avoid the development of resistance to BRAFi. Recent investigations suggest that there are multiple mechanisms responsible for the establishment of resistance to BRAFi, which can be grouped into two major modes MEK-dependent and MEK-independent [13,14]. In the first and more frequent case, reactivation of the MAPK pathway occurs, for example through the acquisition of novel N-RAS mutations or V600E BRAF truncations resulting in RAS-independent RAF dimerization with other members of the same family. In the second case cancer growth depends upon activation of signaling pathways redundant to MAPK, for example via overexpression of RTKs, such as PDGFR or IGF1R, which promote activation of the PI3K-AKT pathway. These mechanisms have been observed both in melanoma cell cultures exposed in vitro to continuous selection with BRAF inhibitors, and in post-relapse human melanoma tumor samples [14,15]. Importantly, secondary mutations in V600E BRAF have not been identified in drug-resistant tumors, thus arguing that the strategy to overcome BRAFi resistance in melanoma has to rely on the development of combinatorial approaches.

The evidence that resistance to BRAFi frequently depends upon reactivation of the MAPK pathway has led to the development of novel strategies directed to simultaneously co-target BRAF and MEK in the attempt to mitigate the emergence of resistance [15]. Indeed, MEK inhibitors have been shown to increase progression free survival when delivered in combination with a BRAF inhibitor as compared to BRAF inhibitor monotherapy [16]. However, even if combinatorial treatment with BRAFi and MEKi gives rise to increase in time to progression as compared to BRAFi monotherapy, this approach is unable to completely eradicate disease, most likely because of MAPK-independent adaptive changes taking place in melanoma cells upon exposure to inhibitors of this pathway. Therefore additional approaches are under study which include for example combination treatments with MEK and IGF1R/PI3K inhibitors [15].

The EGFR family of receptor tyrosine kinases consists of four closely related family members: EGFR (Her1), ErbB2 (HER2), ErbB3 (Her3), and ErbB4 (Her4) [17]. These receptors are important regulators of normal growth and cell differentiation. Their gene amplification, overexpression or mutation is associated with tumor development and poor clinical prognosis in most of the human cancers [18]. EGFR and HER2, have been among the most extensively studied for the therapy of cancer over the past twenty years and a wealth of drugs directed against them have been either already approved or are in advanced clinical development [18]. Although ErbB3 has been disregarded for several years as a target for cancer therapies recent meta-analysis of ErbB3 expression in several solid tumor demonstrated that increased levels of receptor are constantly associated with worse survival [19]. Indeed, during the last years several evidences have been accumulated pointing to a key role of this receptor in tumorigenesis and cancer progression both as a node in ligand-induced signalling by members of the EGFR family [20] and as a major contributor of resistance to therapies in lung and breast cancer [21-23], which fueled efforts towards the development of ErbB3 inhibitors [24]. We have recently shown that melanoma cells often express ErbB3 in concert with other ErbBs and that neuregulin, acting through ErbB3, activates the PI3K/AKT pathway, thus leading to increased cell survival, proliferation and migration [25]. Furthermore, we have generated a set of three anti-human ErbB3 monoclonal antibodies (mAbs) in our laboratory A2, A3 and A4 [26]. These antibodies have been characterized biochemically and functionally [25,26]. These studies led to conclude that, at least in melanoma cell cultures, A3 and A4, but not A2 are able to strongly inhibit ligand-induced signalling, proliferation and migration. In the attempt to understand their mechanism of action, we demonstrated through a series of combined approaches that antibody efficacy correlated with the ability to induce receptor internalization, degradation and inhibition of receptor recycling to the cell surface [25].

In the present work we show that ErbB3 is central to a feedback survival loop activated in melanoma cells upon exposure to BRAF and/or MEK inhibitors, that this activation is dependent upon increased production and release of neuregulin by melanoma cells and, most importantly, that antibodies against ErbB3 capable to induce receptor degradation, abolish this loop and strongly potentiate the antitumor efficacy of BRAF and or MEK inhibitors when given in combination.

## Methods

### Cell lines and treatments

Human melanoma cell lines LOX IMVI, MST-L and WM266 were cultured in RPMI supplemented with 10% FBS. To evaluate ErbB3, AKT and ERK 1/2 signaling and neuregulin expression melanoma cells were serum starved for 24 h and treated with vemurafenib and/or GSK1120212 at different doses and times and incubated or not with 20 μg/ml of anti-ErbB3 mAbs A4, A3 or A2 . To determine effects on proliferation melanoma cell lines, seeded at 1*10^5^/ well, were treated with increasing concentrations (from 0,002 to 1 μM) of vemurafenib and/or GSK1120212 alone or in combination with anti-ErbB3 mAbs for 10 days. To evaluate neuregulin (HRG) release LOX IMVI cells were treated for 24 h with vemurafenib and then the conditioned medium of BRAFi treated cells (pre-incubated or not with the anti-HRG antibody) was used to stimulate starved LOX IMVI cells for 1 h.

### Antibodies and reagents

Antibodies against AKT, ERK 1/2, phospho-ErbB3, phospho-AKT and phospho-ERK 1/2 were purchased from Cell Signaling Technology. Anti-ErbB3, anti-HRG and anti-GAPDH were obtained from Santa Cruz Biotechnology. Anti-rabbit and anti-mouse were purchased from AbCam. The anti-HRG (blocking peptide) was purchased from Thermo Scientific. Anti ErbB3 antibodies A2, A3 and A4 have been described previously by our laboratory [25,26]. The three anti-ErbB3 antibodies are all of the IgG1 isotype (EM unpublished observation). Vemurafenib and GSK1120212 were obtained from Selleck Chemicals. TaqMan probes for HRG and housekeeping gene 18S were purchased from Applied Biosystems.

### Phospho-RTK array

A human phospho-RTK array (R&D Systems) was used to detect simultaneously the phosphorylation status of RTKs (n = 49) in melanoma cells. Membranes were incubated with cell lysates (100 μg) overnight according to the manufacturer' s protocol. After washing, the membranes were incubated with a phosphotyrosine antibody conjugated to horseradish peroxidase to allow the detection of captured RTKs that are phosphorylated. Array data on images were analyzed using Photoshop Quantity One Program (Bio-Rad LaboratoriesGmbH). Duplicate dots in each corner are positive controls.

### Western blot analysis

Melanoma cells were lysed with RIPA buffer; 50 μg of total protein were resolved under reducing conditions by 8% SDS-PAGE and transferred to reinforced nitrocellulose (BA-S 83, Schleider and Schuell, Keene, NH, USA). The membranes were blocked with 5% non fat dry milk in PBS 0.1% Tween 20, and incubated with the different primary antibodies. The membranes were rehydrated and probed again with anti-GAPDH, to estimate the protein equal loading. Densitometric analysis was performed using Quantity One Program (Bio-Rad Laboratories GmbH) and results were expressed as mean values from three independent experiments.

### RNA extraction and real-time PCR analysis

RNA was extracted using TRIzol method (Invitrogen) according to manufacturer’s instruction and eluted with 0,1% diethylpyrocarbonate (DEPC)- treated water. Total RNA was quantitated by spectrophotometry. Real Time-PCR was assayed by TaqMan® Gene Expression Assays (Applied Biosystems, Foster City, CA). To normalize the amount of source RNA, 18S transcript from the same sample was measured and used as internal reference. Each targeted transcript was validated using the comparative Ct method for relative quantification (∆∆Ct) reference to the amount of a common reference gene (18S). The fold difference was calculated using the comparative ∆Ct and results were reported as mean values from three independent experiments.

### *In vitro* colony formation assay

Cells viability was determined by crystal violet staining. Briefly, the cells were stained for 20 min at room temperature with staining solution (0,5% crystal violet in 30% methanol), washed four times with water and then dried. Cells were then dissolved in a Methanol/SDS solution and the adsorbance (595 nm) was read using a microplate ELISA reader.

### Statistical analysis

Quantitative analyses for curve fitting and for IC50 evaluation, were performed by KaleidaGraph software. p-values were calculated using Student’s t test and significance level has been defined as p < 0,05.

## Results and discussion

### ErbB3 is the only RTK rapidly phosphorylated upon exposure of BRAF mutated melanoma cells to vemurafenib

**Figure 1:**
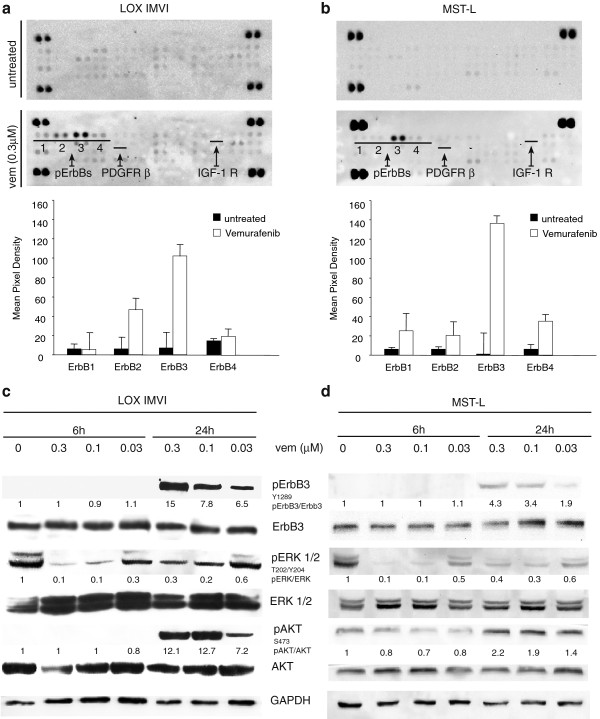
**Vemurafenib treatment induces selective ErbB3 phosporylation in melanoma cells.** Simultaneous detection of the phosphorylation status of RTKs (n = 49) using a human phospho-RTK array in LOX IMVI **(a)** and MST-L **(b)** melanoma cells treated or not for 24 h with 0.3 μM vemurafenib. Membranes were incubated with cell lysates (100 μg) overnight according to the manufacturer' s protocol. The array detects the tyrosine-phosphorylated RTKs simultaneously in duplicate (1, pErbB1; 2, pErbB2; 3, pErbB3; 4, pErbB4). Duplicate dots in each corner are positive controls. Array data on images were analyzed using Photoshop Quantity One Program (Bio-Rad LaboratoriesGmbH). The phosphorylation of ErbB3 is strongly increased by vemurafenib treatment. LOX IMVI **(c)** and MST-L **(d)** cells were serum starved for 24 h, treated or not with different doses of vemurafenib for 6 h or 24 h. Western blot analysis performed using the indicated antibodies shows that vemurafenib induces a strong dose-dependent and time-dependent phosphorylation of ErbB3 and AKT. For densitometric analysis pErbB3/ErbB3, pERK/ERK and pAKT/ATK values are expressed as fold change with respect to the control unstimulated cells to which value = 1 was assigned. Results are reported as mean values ± standard deviation (SD) from three independent experiments.

In order to identify the mechanism responsible for early adaptive changes of melanoma cells to BRAF inhibition, we postulated that receptor tyrosine kinases may be important sensors. Hence, we utilized an RTK array to detect early changes in the phosphorylation level of approximately fifty RTKs. LOX IMVI melanoma cells bearing the most frequent oncogenic BRAF mutation V600E [27] were treated for 24 h with 0.3 μM vemurafenib. Surprisingly we found that, while the phosphorylation level of most receptors remained unchanged or was subjected to subtle variations, the only receptor whose phosphorylation was consistently upregulated 50–100 fold was ErbB3 (Figure 1a). These results were confirmed in two other melanoma cell lines, MST-L [25] bearing a V600R mutation (Figure 1b) and WM266 bearing a V600D [27] mutation (Additional file 1: Figure S1a). Hence, ErbB3 is the major RTK undergoing hyperphosphorylation upon BRAF inhibition in melanoma cells bearing distinct BRAF mutations as well as different ErbB receptor compositions (Additional file 2: Table S1). This strongly suggests that this is a general phenomenon taking place in melanoma when BRAF is inhibited.

Cell extracts of melanoma cell lines LOX IMVI and MST-L exposed to vemurafenib at different doses and times were prepared and subjected to western blotting. The results **(**Figure 1c and d) show that ErbB3 undergoes a strong dose- and time-dependent upregulation of its phosphorylation in the absence of external addition of neuregulin (HRG). Feedback activation of pErbB3 was accompanied by increased phosphorylation of AKT (Figure 1c and d), which suggests the activation of a pro-survival loop contributing to dampen the efficacy of BRAF inhibitors. Importantly the same findings were confirmed in WM266 **(**Additional file 1: Figure S1b**)**. It is important to point out that pErbB3 upregulation takes place in the absence of increased levels of ErbB3 protein (see WBs in Figure 1c and d and Additional file 1: Figure S1b) and in the absence of increased levels of ErbB3 and FOXD3 mRNA as indicated by gene expression profiling of untreated *vs* vemurafenib treated melanoma cells (not shown).

### BRAF inhibitor-induced feedback survival loop is abrogated by anti-Erbb3 antibodies

**Figure 2:**
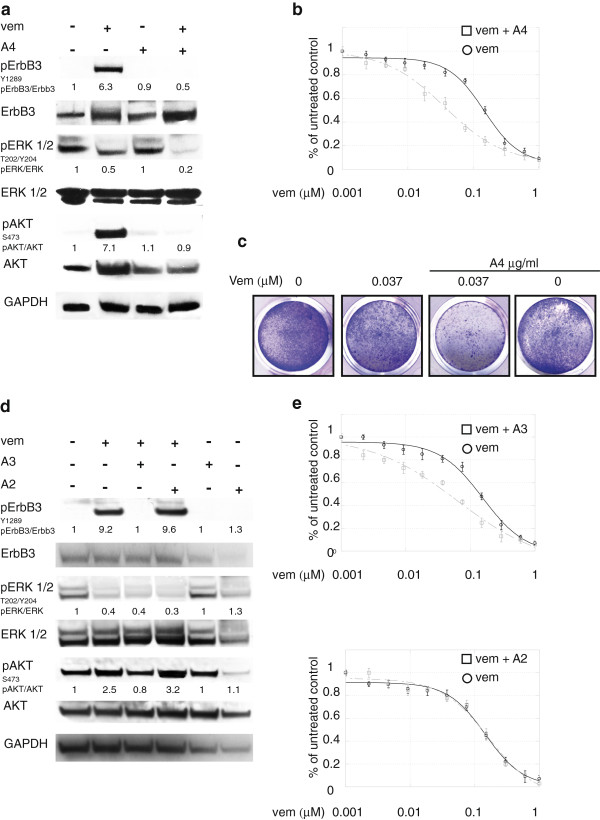
**Anti-ErbB3 mAbs differently counteract the increase of ErbB3-dependent AKT phosphorylation and potentiate growth inhibition induced by vemurafenib.** LOX IMVI melanoma cells serum starved and treated with vemurafenib (0.3 μM) for 24 h were incubated or not with 20 μg/ml of anti-ErbB3 mAbs A4 **(a)**, A3 or A2 **(d)**. Western blot analysis shows that A4 and A3, but not A2 mAbs abrogate receptor phosphorylation and ATK signaling. For densitometric analysis pErbB3/ErbB3, pERK/ERK and pAKT/ATK values are expressed as fold change with respect to the control unstimulated cells to which value = 1 was assigned. Results are expressed as mean values from three independent experiments. LOX IMVI cells were grown in the presence of different doses of vemurafenib alone or in combination with 20 μg/ml of A4 **(b)**, A3 or A2 **(e)** mAbs for 10 days. Cells were then fixed and stained with crystal violet **(c)**. Cells were then dissolved in a Methanol/SDS solution and the adsorbance (595 nm) was read using a microplate ELISA reader **(b, e)**. Quantitative analysis for curve fitting and for IC50 evaluation, performed by KaleidaGraph software, shows that the treatment with A4 and A3 but not with A2 enhances the inhibitory effect of vemurafenib on cell growth (IC50 vem = 155 nM; IC50 vem + A4 = 36 nM; IC50 vem + A3 = 62, IC50 vem + A2 = 146 nM). Results are reported as mean values ± standard deviation (SD) from three independent experiments. p-values were calculated using Student’s t test and significance level has been defined as p < 0,05. For IC50 vem + A4 and IC50 vem + A3 p < 0,001 vs IC50 vem; IC50 vem + A2 NS vs IC50 vem.

We therefore assessed the effect of the anti-ErbB3 antibody A4 generated in our lab and able to inhibit the ligand-induced signaling and to potently induce receptor internalization and degradation [25,26], on vemurafenib-induced pErbB3 and pAKT levels and found that this was able to completely abrogate receptor phosphorylation and AKT signaling in all cell lines tested (Figure 2a**,** for LOX IMVI and Additional file 3: Figure S2a and c for MST-L and WM266 respectively). Also, it is important to notice that the combined treatment led to a stronger degree of pERK down regulation.

In order to assess whether inhibition of pErbB3 and pAKT could result in potentiation of the growth inhibitory effects of vemurafenib, *in vitro* colony formation assays were carried out in the presence of growing concentrations of vemurafenib alone or in combination with a fixed dose of A4. Remarkably, treatment with anti-ErbB3 mAb strongly potentiated growth inhibition by vemurafenib (Figure 2b and c for LOX IMVI and Additional file 3: Figure S2b and d for MST-L and WM266 respectively). In order to further confirm the specificity of this effect we tested in LOX IMVI cells two other anti-ErbB3 mAbs from our collection, namely A3 and A2 which were previously shown to be able to inhibit or not ErbB3-dependent signaling respectively [25]. As expected only A3 but not A2 was able to completely abrogate vemurafenib-induced ErbB3 phosphorylation and AKT signaling (Figure 2d). Moreover in *in vitro* colony formation assays only A3 but not A2 strongly potentiated growth inhibition by vemurafenib (Figure 2e).

### The ErbB3 feedback survival loop is activated also upon MEK inhibition

The evidence that one of the most frequent mechanisms responsible for the development of stable resistance to BRAF is reactivation of the MAPK/ERK pathway has driven the clinical development of MEK inhibitors [14,16].

**Figure 3:**
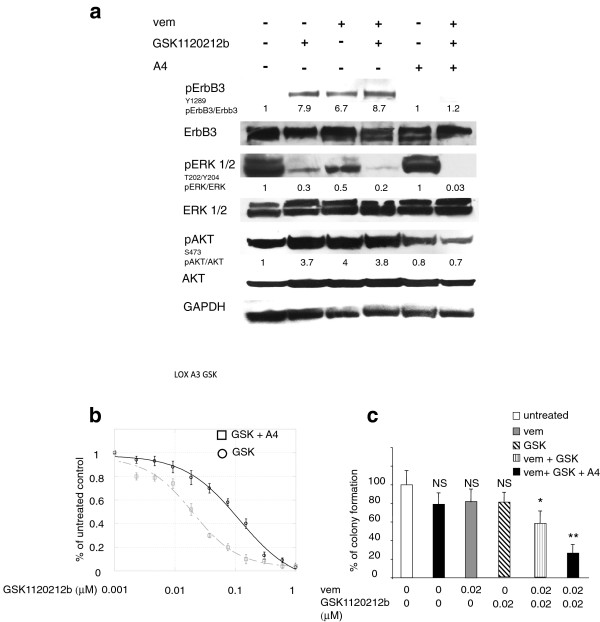
**Anti-ErbB3 mAb A4 counteracts the increase of ErbB3-dependent AKT phosphorylation and potentiate growth inhibition induced by GSK1120212b. (a)** LOX IMVI melanoma cells were serum starved and treated with vemurafenib (0.3 μM), with GSK1120212b (GSK, 0.15 μM) or with their combination in presence or not of anti-ErbB3 mAb A4 (20 μg/ml) for 24 h. Western blot analysis shows that A4 mAb abrogate ErbB3 phosphotylation as well as the strong increase of pAKT induced by both inhibitors**.** For densitometric analysis pErbB3/ErbB3, pERK/ERK and pAKT/ATK values are expressed as fold change with respect to the control unstimulated cells to which value = 1 was assigned. Results are expressed as mean values from three independent experiments. **(b)** Cells were grown in the presence of different doses of GSK combinated or not with A4 mAb (20 μg/ml) for 10 day. Cells were then dissolved in a Methanol/SDS solution and the adsorbance (595 nm) was read as above. Quantitative analysis for curve fitting and for IC50 evaluation, performed as above, shows that the treatment with A4 enhances the inhibitory effect of GSK on cell growth (IC50 GSK = 115 nM; IC50 GSK + A4 = 19 nM). p-values were calculated and significance level has been defined as above. For IC50 GSK + A4 p < 0,001 vs IC50 GSK. **(c)** Cells were treated with suboptimal doses of vemurafenib, GSK or their combination in presence or not of A4 mAb **(c)**. The *in vitro* colony formation assay shows that the addition of A4 significantly inhibits cells growth. *p < 0,01 vs vem-treated or GSK-treated cells; ** p < 0,001 vs vem + GSK- treated cells; NS vs untreated cells.

We have, therefore, investigated whether the ErbB3-dependent feedback survival loop is activated also by MEK inhibitors. To this purpose we treated LOX-IMVI cells with GSK1120212b. As it is shown in Figure 3a and Additional file 4: Figure S3, a strong induction of pErbB3, with concomitant increase of pAKT was observed 24 h after cell exposure to the MEK inhibitor. Also in this case the feedback survival loop was fully abrogated by the addition of the anti-ErbB3 mAb A4 (Figure 3a). *In vitro* colony formation assays were run in the presence of growing concentrations of GSK1120212b alone or in combination with a fixed dose of A4. Also in this case, co-treatment with anti-ErbB3 strongly potentiated growth inhibition by the MEK inhibitor (Figure 3b). Our results clearly indicate that targeting of the RAS-RAF-MAPK pathway at multiple levels is unable to avoid bypass activation of the AKT-dependent adaptive mechanism centered around ErbB3, and that cell treatment with anti-ErbB3 has a dominant effect on both pAKT and pERK when combined with a BRAF and MEK inhibition. Finally when cells were treated with suboptimal doses of vemurafenib and GSK1120212b, the addition of A4 was capable to provide a powerful synergistic inhibition of cell growth (Figure 3c).

### The feedback survival loop is promoted by increased autocrine production of neuregulin by melanoma cells

**Figure 4:**
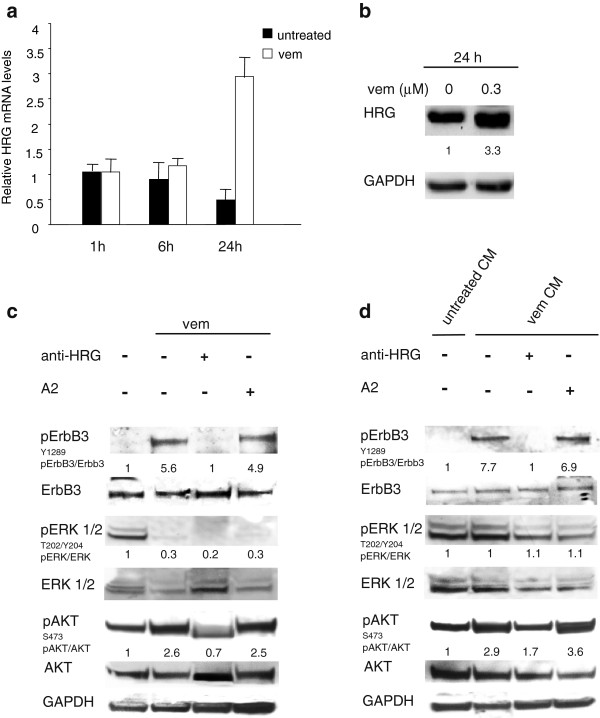
**Vemurafenib treatment induces increased expression and release of neuregulin (HRG) in LOX IMVI melanoma cells.** Cells were serum starved for 24 h and treated or not with vemurafenib (0,3 μM) for 1, 6 or 24 h. Real-time PCR analysis performed using specific TaqMan probes **(a)** and Western blot analysis performed using anti-HRG antibody **(b)** show that upon 24 h of vemurafenib treatment HRG expression is increased at both mRNA and protein levels. For PCR and Western Blot analyses results are reported as mean values ± standard deviation (SD) from three independent experiments. **(c)** Cells were treated with vemurafenib in presence or not of a neutralizing anti-HRG antibody. The Anti-ErbB3 A2 mAb was used as negative control. The anti-HRG antibody, but not A2 mAb, strongly inhibits both ErbB3 and AKT phosphorylation triggered by vemurafenib. **(d)** Cells treated for 1 h with the conditioned medium (CM) from untreated-LOX IMVI cells (untreated CM) or from vemurafenib-stimulated LOX IMVI cells (vem CM) were pre-incubated or not with the anti-HRG antibody or with A2 mAb. The treatment with the anti-HRG antibody, but not A2 mAb abrogates both ErbB3 and AKT phosphorylation induced by vem CM. For densitometric analysis pErbB3/ErbB3, pERK/ERK and pAKT/ATK values are expressed as fold change with respect to the control unstimulated cells to which value = 1 was assigned. Results are expressed as mean values from three independent experiments.

We were interested to better dissect the molecular mechanism responsible for drug-dependent pErbB3 upregulation. Normally, ErbB3 is phosphorylated following ligand-dependent hetero-dimerization with other HER-family receptor partners. Since BRAF or MEK inhibitors induce pErbB3 in melanoma cells bearing different HER-family receptor composition (see Additional file 2: Table S1) we reasoned that a common mechanism could be the increased production and release of neuregulin in the medium and activation of an autocrine loop. Indeed, real time PCR analysis of LOX IMVI cells treated at different times with vemurafenib showed increased neuregulin mRNA levels 24 h after treatment (Figure 4a). This was accompanied by increased production of neuregulin as detected by western blotting (Figure 4b). To confirm that the autocrine production of neuregulin is responsible for the ErbB3-mediated survival loop in response to BRAF inhibitor LOX IMVI cells were treated with vemurafenib in presence or not of a neutralizing antibody against neuregulin (Figure 4c). Western blot analysis clearly showed that the anti-HRG antibody strongly inhibit both pErbB3 and consequently pAKT. To further confirm this, the conditioned medium of BRAFi treated melanoma cells was able to induce pErbB3 in starved melanoma cells with a very rapid kinetic, after 1 hour exposure (Figure 4d). Finally in order to fully prove that this mechanism is entirely dependent upon increased production of the ligand, the conditioned medium was pre-incubated with neutralizing antibodies against neuregulin. As shown in Figure 4d**,** this treatment fully abrogated pErbB3 induction by the conditioned medium of drug-treated melanoma cells.

## Conclusions

A current limitation of targeted therapies against metastatic melanoma with BRAF or MEK inhibitors is the development of resistance. Hence it is of utmost importance to identify and tackle the underlying mechanisms. Nazarian et al. [14] and Villanueva et al. [15] have previously reported that acquired resistance to vemurafenib, which develops after prolonged exposure to the drug *in vitro* or *in vivo*, is caused by mutually exclusive mechanisms: either mutations in N-RAS or up regulation of PDGFR/ IGF1R signaling. These changes are presumably the resultant of a prolonged process preceded by adaptive changes which allow cells to survive while long-term stable resistant are selected. In the present work we have decided to focus our attention on these early adaptive changes in order to identify the major pathways involved. As first approach we carried out an RTK array to identify receptor tyrosine kinases undergoing variations in their phosphorylation after short-term exposure to vemurafenib. Surprisingly, we found that in the three melanoma cell lines tested, the only receptor which underwent prominent hyperphosphorylation was ErbB3. It has to be pointed out that in this early phase, at least in the cell lines used in our study, both PDGFR and IGF1R didn’t modify their level of phosphorylation, which leads us to conclude that the involvement of these receptors may occur only at a later time when long-term resistant clones are selected [14,15].

Abel et al. have recently reported that melanoma cells adapt to RAF/MEK inhibitors through a FOXD3-mediated upregulation of ErbB3 transcription [28], which induced cell sensitization to the biological effect of exogenously added ErbB3 ligand neuregulin. A similar involvement of ErbB3 was also previously suggested by Lito et al. [7]. Our biochemical findings are entirely novel and substantially differ from the previous ones. The major difference is that, while Abel et al. attribute the involvement of ErbB3 to increased FOXD3-dependent ErbB3 gene transcription upon exposure to vemurafenib, which leads to a moderate 2-fold increase in ErbB3 protein levels, we do not detect major variations in total ErbB3 protein levels upon exposure to either a BRAF or to a MEK inhibitor. In contrast we detect a prominent (several-fold) spontaneous hyperphosphorylation of the receptor consequent to the increased cell production and release of neuregulin with consequent activation of an autocrine loop. We cannot easily explain these significant discrepancies. However it is worth pointing out that Abel et al. use melanoma cells stably transfected with a plasmid encoding Foxd3, and which therefore express supraphysiological amounts of this transcription factor [28]. In contrast we never transfect this factor and, therefore, we believe we work in more physiological conditions.

In addition we show for the first time that neutralizing antibodies against ErbB3 are capable to fully abrogate this compensatory survival mechanism and to potently synergize with BRAF and MEK inhibitors. Therefore, we propose that initial co-treatment of melanoma patients bearing BRAF mutations with an anti-ErbB3 antibody could be a powerful strategy to enhance clinical efficacy of BRAF and MEK inhibitors.

## Abbreviations

MAPK: Mitogen-activated protein kinase; Vem: Vemurafenib; GSK: GSK1120212b; HRG: Neuregulin; MAbs: Monoclonal antibodies.

## Competing interests

Dr Paolo A. Ascierto participated to Advisory Board from Bristol Myers Squibb, MSD, Roche Genentech, GSK, and received honoraria from Brystol Myers Squibb, MSD and Roche-Genentech. All remaining authors declare the absence of any competing interest.

## Authors’ contributions

LF performed data acquisition, data analysis, preparation of the illustrations, and drafted the manuscript; MEP, AN and CDV contributed to acquisition and analysis of western blotting and cell proliferation data; EM, FB, LA and RM contributed to data analysis and draft of sections of the manuscript; MRT and PAA provided melanoma cells lines, continuous advice to the study and revised the manuscript; GC conceived and supervised the study, and revised the manuscript. All authors read and approved the final version of the manuscript.

## Supplementary Material

Additional file 1: Figure S1 Vemurafenib treatment induces selective ErbB3 phosphorylation in WM266 melanoma cells. **(a)** Simultaneous detection of the phosphorylation status of RTKs (n = 49) using a human phospho-RTK array in WM266 melanoma cells treated or not for 24 h with 0.3 μM vemurafenib. Membranes were incubated with cell lysates and array data were analyzed as reported in Figure 1. The phosphorylation of ErbB3 is strongly increased by vemurafenib treatment. **(b)** WM266 cells were serum starved for 24 h, treated or not with different doses of vemurafenib for 6 h or 24 h. Western blot analysis shows a strong dose-dependent and time-dependent phosphorylation of ErbB3 and AKT induced by vemurafenib. For densitometric analysis pErbB3/ErbB3, pERK/ERK and pAKT/ATK values are expressed as fold change with respect to the control unstimulated cells to which value = 1 was assigned. Results are reported as mean values ± standard deviation (SD) from three independent experiments. Click here for file

Additional file 2: Table S1 Flow cytometry analysis of ErbBs membrane expression in LOX IMVI, MST-L and WM266 melanoma cell lines. The percentage of positive cells was determined by staining with the indicated primary antibodies and with the isotype-matched andibodies as negative control. LOX IMVI, MST-L and WM266 show different ErbB receptor compositions. Click here for file

Additional file 3: Figure S2 Anti-ErbB3 A4mAb counteracts the increase of ErbB3-dependent AKT phosphorylation and potentiate growth inhibition induced by vemurafenib in melanoma cells. MST-L **(a)** and WM266 **(c)** cells serum starved and treated with vemurafenib (0.3 μM) for 24 h were incubated or not with A4 mAb (20 μg/ml). Western blot analysis shows that A4 abrogate receptor phosphorylation and ATK signaling. For densitometric analysis pErbB3/ErbB3, pERK/ERK and pAKT/ATK values are expressed as fold change with respect to the control unstimulated cells to which value = 1 was assigned. Results are expressed as mean values from three independent experiments. MST-L **(b)** and WM266 **(d)** cells were grown in the presence of different doses of vemurafenib alone or in combination with a fixed dose (20 μg/ml) of A4. Cells were then dissolved in a Methanol/SDS solution and the adsorbance (595 nm) was read as reported in Figure 2. Quantitative analysis for curve fitting and for IC50 evaluation, performed as reported in Figure 2, shows that A4 enhances the inhibitory effect of vemurafenib on both cell lines’ growth (for MST-L cells: IC50 vem = 264 nM, IC50 vem + A4 = 69 nM; for WM266 cells IC50 vem = 140 nM, IC50 vem + A4 = 51 nM). Results are reported as mean values± standard deviation (SD) from three independent experiments. p-values were calculated and significance level has been defined as reported in Figure 2. For MST-L and WM266 cells IC50 vem + A4 p < 0,001 vs IC50 vem. Click here for file

Additional file 4: Figure S3 GSK1120212b treatment induces selective ErbB3-dependent AKT phosphorylation in LOX IMVI melanoma cells. Cells were serum starved for 24 h, treated or not with different doses of GSK for 6 h or 24 h. Western blot analysis performed using the indicated antibodies shows that GSK induces a strong dose-dependent and time-dependent phosphorylation of ErbB3 and AKT. For densitometric analysis pErbB3/ErbB3, pERK/ERK and pAKT/ATK values are expressed as fold change with respect to the control unstimulated cells to which value = 1 was assigned. Results are expressed as mean values from three independent experiments. Click here for file
