# Supplementary figures and images for: Activation of an early feedback survival loop involving phospho-ErbB3 is a general response of melanoma cells to RAF/MEK inhibition and is abrogated by anti-ErbB3 antibodies
Source: J Transl Med. 2013 Jul 27;11:180. doi: 10.1186/1479-5876-11-180 (PMC3729364; doi:10.1186/1479-5876-11-180)

**a**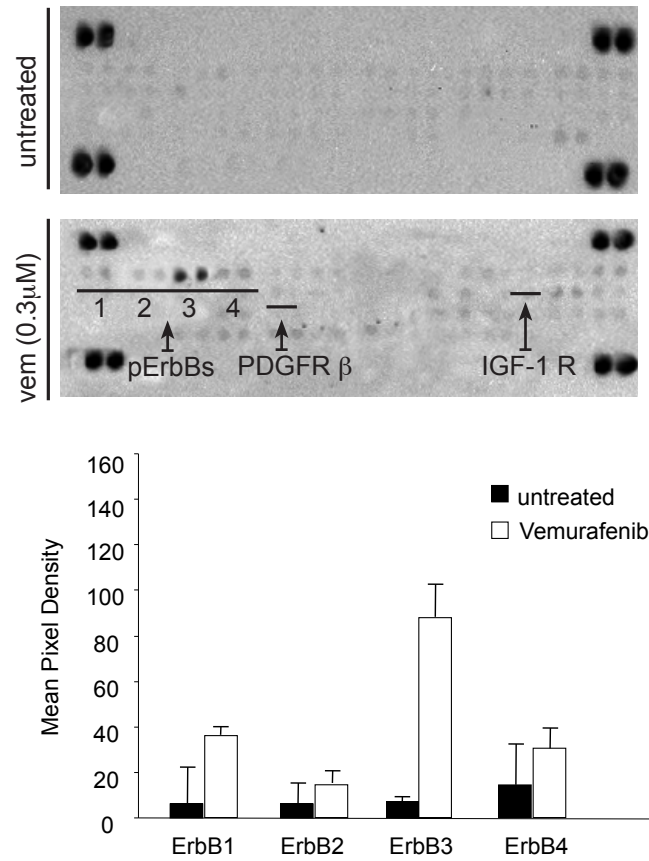**b**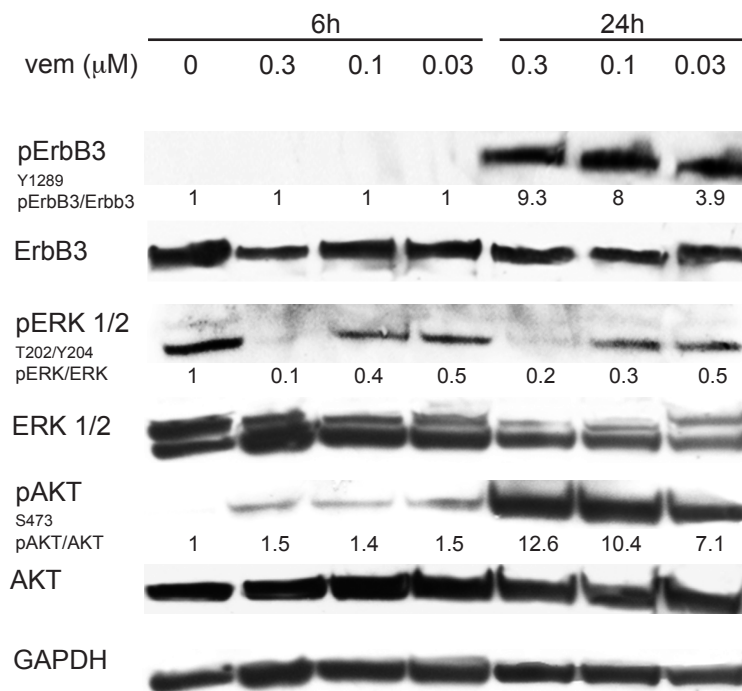**Suppl. Fig.1**

Supplement: Additional file 1: Figure S1 — Vemurafenib treatment induces selective ErbB3 phosphorylation in WM266 melanoma cells. (a) Simultaneous detection of the phosphorylation status of RTKs (n = 49) using a human phospho-RTK array in WM266 melanoma cells treated or not for 24 h with 0.3 μM vemurafenib. Membranes were incubated with cell lysates and array data were analyzed as reported in Figure 1. The phosphorylation of ErbB3 is strongly increased by vemurafenib treatment. (b) WM266 cells were serum starved for 24 h, treated or not with different doses of vemurafenib for 6 h or 24 h. Western blot analysis shows a strong dose-dependent and time-dependent phosphorylation of ErbB3 and AKT induced by vemurafenib. For densitometric analysis pErbB3/ErbB3, pERK/ERK and pAKT/ATK values are expressed as fold change with respect to the control unstimulated cells to which value = 1 was assigned. Results are reported as mean values ± standard deviation (SD) from three independent experiments. [file 1479-5876-11-180-S1.pdf]

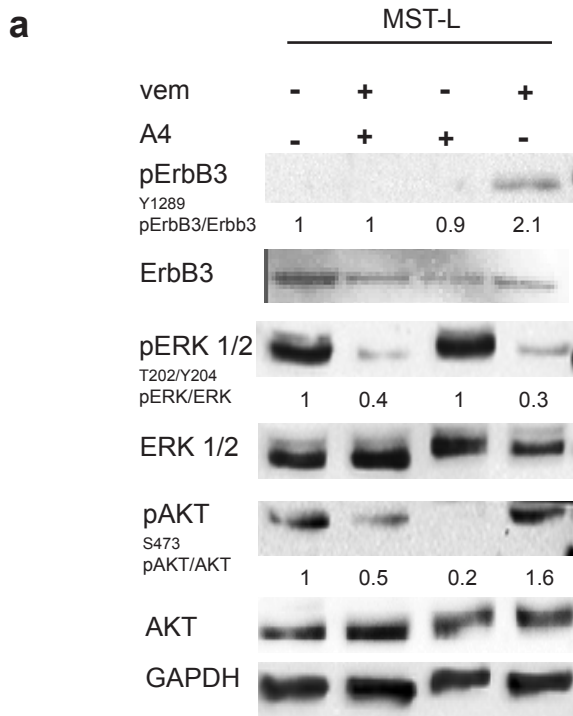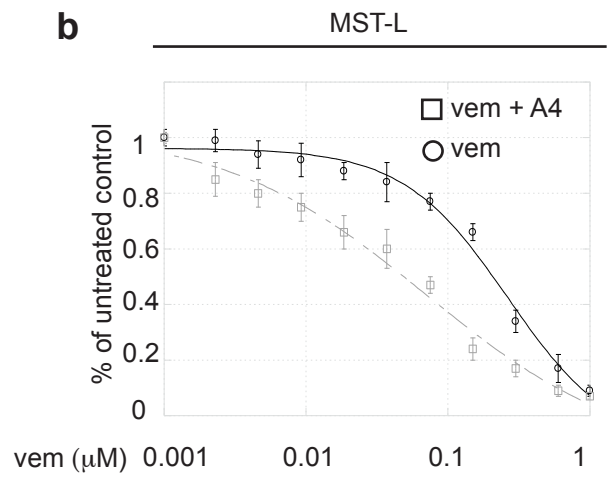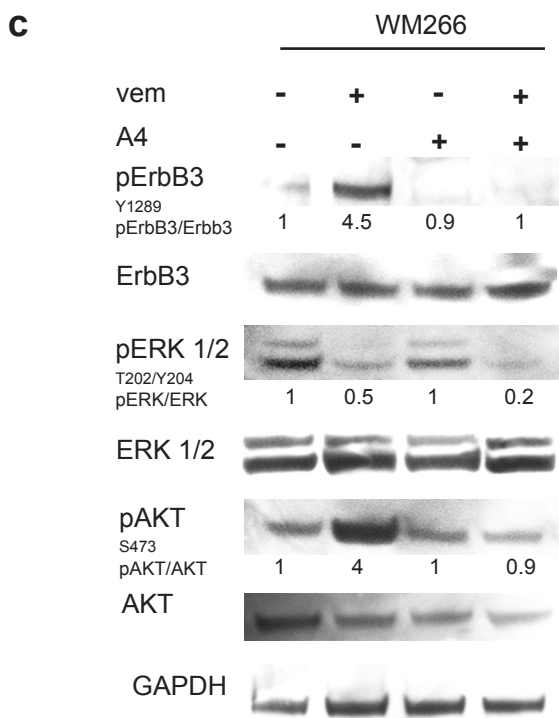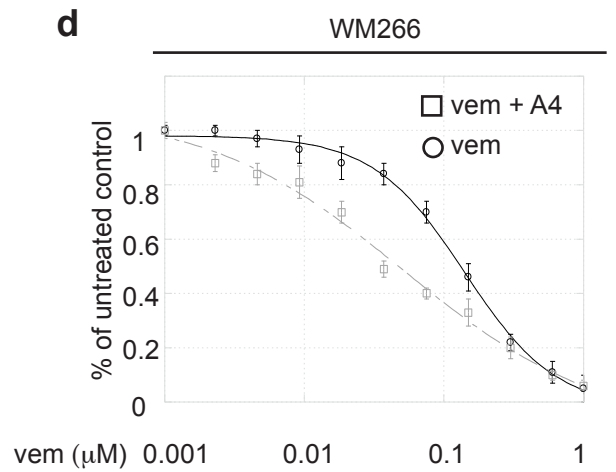

Suppl. Fig.2

Supplement: Additional file 3: Figure S2 — Anti-ErbB3 A4mAb counteracts the increase of ErbB3-dependent AKT phosphorylation and potentiate growth inhibition induced by vemurafenib in melanoma cells. MST-L (a) and WM266 (c) cells serum starved and treated with vemurafenib (0.3 μM) for 24 h were incubated or not with A4 mAb (20 μg/ml). Western blot analysis shows that A4 abrogate receptor phosphorylation and ATK signaling. For densitometric analysis pErbB3/ErbB3, pERK/ERK and pAKT/ATK values are expressed as fold change with respect to the control unstimulated cells to which value = 1 was assigned. Results are expressed as mean values from three independent experiments. MST-L (b) and WM266 (d) cells were grown in the presence of different doses of vemurafenib alone or in combination with a fixed dose (20 μg/ml) of A4. Cells were then dissolved in a Methanol/SDS solution and the adsorbance (595 nm) was read as reported in Figure 2. Quantitative analysis for curve fitting and for IC50 evaluation, performed as reported in Figure 2, shows that A4 enhances the inhibitory effect of vemurafenib on both cell lines’ growth (for MST-L cells: IC50 vem = 264 nM, IC50 vem + A4 = 69 nM; for WM266 cells IC50 vem = 140 nM, IC50 vem + A4 = 51 nM). Results are reported as mean values± standard deviation (SD) from three independent experiments. p-values were calculated and significance level has been defined as reported in Figure 2. For MST-L and WM266 cells IC50 vem + A4 p < 0,001 vs IC50 vem. [file 1479-5876-11-180-S3.pdf]

**a**

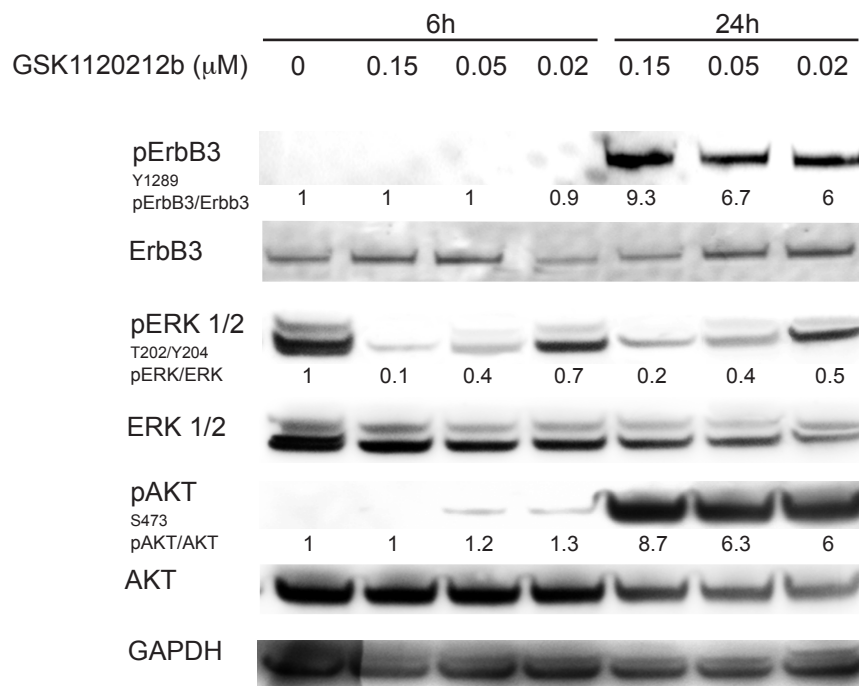

**Suppl. Fig.3**

Supplement: Additional file 4: Figure S3 — GSK1120212b treatment induces selective ErbB3-dependent AKT phosphorylation in LOX IMVI melanoma cells. Cells were serum starved for 24 h, treated or not with different doses of GSK for 6 h or 24 h. Western blot analysis performed using the indicated antibodies shows that GSK induces a strong dose-dependent and time-dependent phosphorylation of ErbB3 and AKT. For densitometric analysis pErbB3/ErbB3, pERK/ERK and pAKT/ATK values are expressed as fold change with respect to the control unstimulated cells to which value = 1 was assigned. Results are expressed as mean values from three independent experiments. [file 1479-5876-11-180-S4.pdf]
