# Supplementary material for: Activation of an early feedback survival loop involving phospho-ErbB3 is a general response of melanoma cells to RAF/MEK inhibition and is abrogated by anti-ErbB3 antibodies
Source: J Transl Med. 2013 Jul 27;11:180. doi: 10.1186/1479-5876-11-180 (PMC3729364; doi:10.1186/1479-5876-11-180)
Supplement: Additional file 2: Table S1 — Flow cytometry analysis of ErbBs membrane expression in LOX IMVI, MST-L and WM266 melanoma cell lines. The percentage of positive cells was determined by staining with the indicated primary antibodies and with the isotype-matched andibodies as negative control. LOX IMVI, MST-L and WM266 show different ErbB receptor compositions. [file 1479-5876-11-180-S2.doc]

| **Cell lines** | **ErbB1**  **expression** | **ErbB2 expression** | **ErbB3 expression** | **ErbB4 expression** |
| --- | --- | --- | --- | --- |
| LOX IMVI (V600E) | 2% | 42% | 71% | 74% |
| MST-L  (V600R) | 1% | 9% | 39% | 48% |
| WM266  (V600D) | 22% | 65% | 68% | 59% |

**LOX IMVI, MST-L and WM266 melanoma cells show different ErbB receptor compositions.**

**Supplementary Table 1.**

Flow cytometry analysis of ErbBs membrane expression in LOX IMVI, MST-L and WM266 melanoma cell lines. The percentage of positive cells was determined by staining with the indicated primary antibodies and with the isotype-matched andibodies as negative control. LOX IMVI, MST-L and WM266 show different ErbB receptor compositions.
